# Selected Trends in Psychotherapy Research: An Index Analysis of RCTs

**DOI:** 10.32872/cpe.7921

**Published:** 2022-06-30

**Authors:** Winfried Rief, Melina Kopp, Roya Awarzamani, Cornelia Weise

**Affiliations:** 1 Division of Clinical Psychology and Psychotherapy, Department of Psychology, Philipps-University of Marburg, Marburg, Germany; Goethe University, Frankfurt, Frankfurt, Germany

**Keywords:** psychotherapy research, randomized clinical trials RCT, CBT, psychodynamic treatments, ACT, eHealth, mindfulness, schema therapy, systemic therapy, mental health care

## Abstract

**Background:**

We wanted to analyze trends in psychotherapy research during the last decade. We used published randomized clinical trials (RCTs) that are cited in Web of Science (WoS) as an index for these activities.

**Method:**

We searched for RCTs published between the years 2010 and 2019. Search criteria included cognitive-behavioral treatments (CBT), e-mental health, Acceptance and Commitment Therapy (ACT), psychodynamic treatments, interpersonal therapy (IPT), schema therapy, systemic therapy, mindfulness treatments, and emotion-focused therapy (EFT). The numbers of publications for each treatment approach were accumulated for 5-year blocks (2010 to 2014; 2015 to 2019).

**Results:**

The search revealed 4,523 hits for the selected treatment options, of which 1,605 were finally included in the analysis. There was a continuous increase in published RCTs, with 68% more trials during the second five-year block. CBT (68%) and eHealth interventions (18%) show an increase in the number of studies, but there were no significant changes in its percentage in relation to all published RCTs. The next frequent treatments were ACT (4%), psychodynamic treatments (2%), IPT (2%), and mindfulness interventions (2%). We found a significant increase of the percentage of mindfulness (p = .008) and a significant decrease of the percentage of psychodynamic treatments (p = .02). Systemic (1.1%), emotion-focused (0.7%) and schema therapy (0.6%) represented smaller parts of published RCTs.

**Conclusion:**

A continuous increase of published RCTs underlines an active field of research on psychological interventions. Third wave treatments such as mindfulness increased their representation in research, while the part of psychodynamic treatments decreased.

## Highlights

- Over the period from 2010 to 2019, the number of randomized clinical trials (RCTs) of psychological treatments continuously increased, with CBT representing the majority of published RCTs.
- The number of trials on eHealth-interventions increased over time, but their percentage in relation to all clinical trials did not increase significantly.
- Third wave interventions either already represented a significant proportion of RCTs (e.g., ACT), or showed significantly increasing numbers (mindfulness interventions).
- More traditional approaches represented very small percentages of RCTs (e.g., systemic treatments), or even showed a significant decreased percentage of all RCT-based research (i.e., psychodynamic therapy).

Evidence based psychotherapy is a dynamic field of research. In particular, the last 30 years were characterized by innovations in the field of psychological treatments. Advances have been made both in terms of newly developed interventions (e.g. “third wave”-therapies like ACT or mindfulness-based interventions (Haller, Breilmann, Schroter, Dobos, & Cramer, 2021; Hayes, Luoma, Bond, Masuda, & Lillis, 2006; Hofmann & Asmundson, 2008; Teasdale et al., 2000); mentalization based therapy (Bateman & Fonagy, 2010; Taubner & Volkert, 2019), and new formats to provide psychological treatment (e.g. using electronic media such as the internet and mobile phones; Andersson et al., 2019; Miloff, Lindner, & Carlbring, 2020). However, clear data proving these trends in terms of research activities (i.e. clinical trials) are lacking. How do the flagships of psychotherapy such as psychodynamic treatments, CBT, and others progress in this continuously changing field? Do they lose terrain to new concepts, or are they able to maintain their positions?

More knowledge about current research trends in psychotherapy is helpful to estimate and predict future developments. It can be postulated that those approaches that are currently under investigation will likely influence the future delivery of psychotherapy in health care systems that are based on empirical evidence (AWMF, 2021; Berry & Haddock, 2008; Clark, 2011, 2018; NHS, 2019). To date, several countries aim to link the provision of psychotherapy to its evidence base; however, there is still a wide range. While some countries provide mental healthcare that is more linked to traditional orientations (e.g. China; Ng et al., 2017), other countries offer (and permit) nearly all orientations of psychotherapy without making a link to their differing evidence base (e.g. Austria; Laireiter & Weise, 2019). A pioneer in this context is England, which tries to implement a fully evidence-based system for psychological therapies, the "Improving Access to Psychological Therapies"-program (IAPT; NHS, 2019). If countries want to move forward with their health care systems in the direction of evidence-based psychological treatments, they need to know current trends and developments in psychotherapy research.

In the German healthcare system we find an example for the interaction between evidence-base and health care regulations. The federal government established a scientific advisory board on psychotherapy ("Wissenschaftlicher Beirat Psychotherapie" [WBP]), that evaluates whether psychotherapeutic approaches are considered as evidence-based for a broad variety of mental disorders. A final positive vote opens the door for the respective treatment to enter a publicly financed health care system. Such a positive statement was given for psychodynamic treatments, systemic treatments and CBT. A recent application for approval of humanistic treatments (including Rogerian psychotherapy) was rejected on the grounds that the quantity of submitted studies were considered insufficient, and the quality criteria of studies did not meet current standards (WBP, 2018). A clear decline of research activities in this field in the 90ies was evident. Humanistic and Rogerian psychotherapy is therefore not a stand-alone treatment of the German public health care system.

The current manuscript reports on a databased analysis of research trends in psychological treatments. While we did not aim to detect all published trials, we focus on the use of a plausible index of publication activities (index approach). We limit our analysis to one of the major global citation databases (i.e. Web of Science, WoS), in which indexed journals have to go through a thorough editorial selection process ensuring sufficient quality of the included journal (e.g. journal must contain primarily original scholarly material). Furthermore, we limit our research to randomized clinical trials (RCT). These results are used as an index of current trends in psychotherapy research. We are aware that these results only indicate trends, and are not a comprehensive summary of all potentially relevant research activities. Our approach is limited to the used search terms, and treatments of interest. We decided to focus on the three traditional and approved treatment for which evidence has been sufficiently proven and which were commonly used in mental health care (psychodynamic, systemic, CBT), to compare them to newer developments such as ACT, mindfulness, IPT, schema therapy, emotion-focused treatments, or eHealth applications. Mentalization-based interventions were grouped with psychodynamic treatments. A specific problem is evident for CBT treatments, although it partly applies to other treatments as well: labels and approaches for one treatment approach can be very diverse, thus preventing them to be covered by search terms (e.g., some textbooks on CBT report up to 100 different techniques). Therefore again, our analysis is only able to reveal indices, but not a complete picture for general trends in psychotherapy research.

## Method

### Search Procedure

We chose the citation database “Web of Science” to search for research activities during the last decade for the following reasons: (1) We wanted to ensure a certain quality of trials. WoS requires indexed journals to provide a minimum of quality criteria (e.g. peer review, content relevance, appropriate citations). (2) WoS is less focused on medical research, and includes more psychological and social science studies than PubMed. It includes all publications of the Science Citation Index and the Social Science Citation Index (Falagas, Pitsouni, Malietzis, & Pappas, 2008). (3) WoS has a strong focus on peer-reviewed journal publications of research studies, while other databases also include conference abstracts or monographies (e.g. Scopus). In a recent analysis exploring the optimal combination of databases needed for a systematic review, WoS had an overall recall rate of 68% (Bramer, Rethlefsen, Kleijnen, & Franco, 2017). Yet, it must be considered that recall rates are topic-sensitive and that we did not aim to conduct a systematic review.

Since exploratory searches revealed publications of the non-clinical field (e.g. systemic approaches to strengthen the impact of a business, or to improve performance in a school-based setting), we selected specific WoS-categories for our search (e.g., “psychology, clinical” or “neurosciences”). The complete list of selected categories as well as the specific search terms are available in the Supplementary Materials).

Language restrictions were not applied to the searches. The search was conducted in November 2020 and was updated in August 2021.

### Eligibility Criteria

Studies were included if they met the following criteria:

- The study reported results of a randomized clinical trial.
- The RCT investigated one or more of the following psychological treatment approaches: cognitive behavior therapy (CBT), psychodynamic treatments, internet-based psychological treatments and other digital approaches using new technologies (eHealth, mHealth, uHealth), mindfulness-based intervention (mindfulness-based stress reduction (MBSR), mindfulness-based cognitive therapy (MBCT)), acceptance and commitment therapy (ACT), interpersonal therapy (IPT), systemic psychological therapy, schema therapy and emotion-focused therapy (EFT).
- The study was published between 2010 and 2019. This criterion was chosen as we were interested in the most recent trends in psychotherapy research.

We included studies on all age groups (e.g. adults, children, adolescents), all clinical indications for psychotherapy and all countries of origin.

### Study Selection

Only articles reporting the major results of the trials were included (i.e. corrections, conference abstracts, comments etc. were excluded to avoid double-counting). In the case of multiple publications of one trial (e.g., post-treatment findings, follow-up data, other secondary analyses), we selected the publication reporting the primary outcomes at post-treatment. eHealth interventions were only counted under this category, but not further according to the conceptual background. The search was conducted stepwise for all treatment approaches, reviewed by two co-authors (MK, RA); weekly consensus meetings took place. In case of uncertainty, the main supervisor (WR) gave advice.

If a study investigated two or more of the above-mentioned treatment approaches in the investigated treatment arms (e.g. CBT versus ACT), the study was counted for both treatments.

Due to their own theoretical background, we did not consider “third wave interventions” as variants of CBT, but counted ACT, mindfulness, schema therapy, IPT etc. as separate groups, without considering them as CBT variants.

### Analyses

Publications were first grouped according to treatment approach, publication year, and national origin of the principal investigators, to enable an examination of potential regional differences. For the first analysis of publication trends and to avoid too small cell numbers, publications were additionally grouped into five-year periods (2010 to 2014, and 2015 to 2019). For each treatment group, we compared the number of publications between these two time blocks using the chi^2^ test. In case of more than an average of ten annual publications per treatment approach, we report both, analyses of five-year blocks and annual number of RCTs. Additionally, the percentage of publications per treatment approach of all publication hits is computed for the five-year blocks. We also computed the determination coefficient *R*^2^ according to Holt (Holt, 2004) and investigated linear trends in the relationship between publication year and number of publications. This analysis did not only focus on observed data, but also provides an estimation of future developments according to times series modeling. All analyses were conducted using IBM SPSS (Version 26.0) (IBM Corp., 2019).

## Results

Table 1 shows the number of hits of the original searches, and the number of finally included trials after checking the inclusion criteria. From the first to the second five-year block of the last decade (i.e. from 2010-2014 to 2015-2019), we found an overall increase in published RCTs in psychotherapy from 598 to 1,007 (increase of 68%). From 2010 to 2019, the annual number of published RCTs (subsumed over all treatments) increased from 67 to 230 (343%).

**Table 1:** Comparison of Search Hits and Finally Included Trials

Treatment	Hits	Finally included
CBT	3081	1094
eHealth	931	294
Psychodynamic treatments	96	53
ACT	140	61
Systemic therapy	86	21
IPT	87	42
Mindfulness-based interventions	72	21
Schema therapy	18	10
EFT	12	9
Total	4523	1605

### Most Frequently Investigated Psychological Treatments

**Figure 1:**
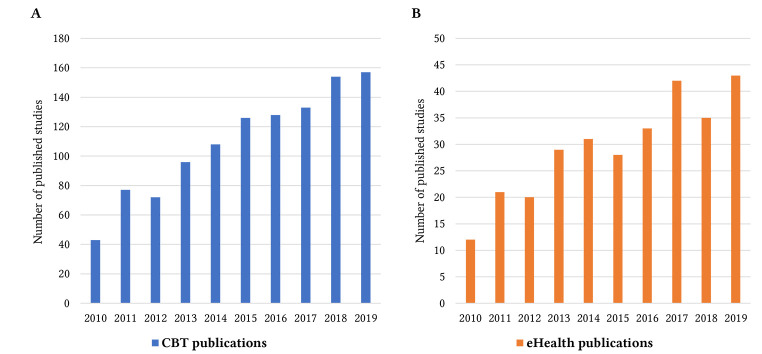
Frequency of Published Studies Including Treatment Arms Testing CBT Interventions (Figure 1A) and eHealth/mHealth Interventions (Figure 1B) Per Year From 2010 to 2019

CBT continues to represent a major part of psychotherapy research with a slight, but non-significant increase from 66% to 68% of all publications comparing the first and the second time block (Table 2). This proportional increase is founded in a more substantial increase in the number of annually published treatment arms using CBT from year to year (see Figure 1a). Considering absolute annual numbers, CBT arms in randomized clinical trials have more than doubled from 2010 to 2019. Holt’s *R*^2^ of .95 indicates that this trend of increasing publications on CBT is highly robust.

**Table 2:** Treatment Arms in RCTs From 2010 to 2019 (Five-Year Blocks)

Treatment Approach	2010–2014	2015–2019	*p* (χ^2^)	*R*^2^ (Holt; prediction per year)
CBT	396 (66.2%)	698 (68.2%)	0.20 (1.52)	.94
eHealth	113 (18.9%)	181 (17.7%)	0.64 (0.21)	.85
ACT	22 (3.7%)	39 (3.8%)	0.84 (0.04)	.62
Psychodynamic treatments	28 (4.7%)	25 (2.4%)	0.02 (5.69)*	-.15
IPT	21 (3.5%)	21 (2.1%)	0.08 (3.00)	-.12
Schema therapy	4 (0.7%)	6 (0.6%)	0.86 (0.03)	-.06
Systemic therapy	10 (1.7%)	11 (1.1%)	0.32 (0.08)	-.17
Mindfulness-based interventions	2 (0.3%)	19 (1.9%)	0.008 (7.02)**	.83
EFT	2 (0.3%)	7 (0.7%)	0.35 (0.88)	.53
Total	598	1007		

*Note*. CBT: cognitive behavior therapy; eHealth: internet-based psychological treatments and other digital approaches using new technologies; ACT: acceptance and commitment therapy; IPT: interpersonal therapy; EFT: emotion-focused treatments. Please note: because of its linear model, Holt’s *R*^2^ can be negative even if five-year block comparisons indicate a significant increase in published treatment arms (e.g., for Schema therapy).

The second most frequently investigated psychological treatment approach is eHealth interventions. However, considering the overall increase of published clinical trials, the proportion of eHealth interventions remained constant from the first to the second five-year period. The increase was based on a continuous increase in published trials on eHealth interventions per year (see Figure 1b) and parallels the growing numbers for psychotherapy trials in general.

For all other types of psychological treatments, the numbers of published trials were not large enough (each less than 5% of all trials) to allow for robust predictions of developments based on annual changes. The specific numbers are listed in Table 1 in the Supplementary Materials.

### Changes From the First to the Second Five-Year Block

After CBT and eHealth interventions, the next most commonly studied treatments are ACT (2015-2019: 4%), psychodynamic treatments (2%), IPT (2%), and mindfulness interventions (2%). We found a significant increase in the percentage of mindfulness interventions (*p* = .008) and a significant decrease in the percentage of psychodynamic treatments (*p* = .02). Systemic therapy (1.1%), emotion-focused treatments (0.7%) and schema therapy (0.6%) represent smaller parts of published RCTs.

Together with systemic therapies, psychodynamic treatments have the highest negative *R*^2^. However, the scores are still very close to zero, indicating that future development is hard to predict.

Although only on a trend level, the situation for IPT seems similar. The number of published treatment arms using this intervention remains stable, but in light of the increasing overall numbers, the proportion of IPT trials is decreasing. Finally, the low number of EFT treatment arms does not allow for any predictions about developments.

### Countries of Origin

Interestingly, the countries of origin of the principal investigator differed depending on the treatment approach. Studies on CBT are dominant in the Anglo-American field (US: 295 treatment arms, UK: 126 treatment arms, Australia: 112 treatment arms). eHealth studies mainly originate from Sweden (67 trials), but also from Australia (48) and Germany (45). Studies on ACT show a strong dominance in the US (20) and Sweden (15). Mindfulness trials originate from many different countries (e.g., US: 4, the Netherlands: 3, and 2 trials each from China, Germany and Iran).

Studies with treatment arms using psychodynamic interventions mainly originate from Germany (21), while rarely coming from other countries (UK: 6; Sweden and Denmark: 5). Finally, IPT trials have a strong dominance in the US (19), with some further activities in China (5) and Germany (4).

## Discussion

With our study, we wanted to investigate indices for research trends. To ensure some basic methodological quality, we limited our search to studies quoted in “Web of Science”, and included only RCTs. Using these specifications, we found a substantial and continuous increase in published research trials on psychotherapy from 2010 to 2019, which more than doubled in this period. Considering the five-year blocks, the increase was 71% in 2015-2019 as compared to 2010-2014. CBT continues to be the most frequently investigated treatment condition, currently representing 68% of treatment arms in RCTs. The increase in CBT studies is quite robust, and statistical predictions indicate that it will continue this way in coming years. eHealth interventions are considered an emerging field in psychotherapy research. Indeed, the total number of published trials continuously increased from 2010 to 2019. The proportion of eHealth interventions in psychotherapy research remained stable. Research activities on third-wave interventions are also very dynamic and characterized by a continuous increase in published trials. Only for mindfulness interventions and schema therapy did we find a significant increase in the proportion of trials in relation to other published RCTs.

The role of the more traditional treatment approaches such as psychodynamic interventions and systemic therapies seems to have continuously decreased. We found significantly smaller proportions of studies that characterized by psychodynamic treatment arms, and a slight (but not significant) decrease in the proportion of systemic treatment arms. In the 2015-2019 period, psychodynamic approaches accounted for only 2.4% of all psychotherapy treatment trials.

Interestingly, the various treatment approaches are differently represented across countries. For example, a large proportion of studies on eHealth interventions originate from Sweden and Australia, whereas CBT treatment arms are dominant in studies from the US. The reasons for these differences can be manifold: regulations of the national health care systems, financial issues imposed by health care providers and pressure for the provision of short-term interventions, the need for cultural adaptation, or regional conditions such as the distance to available health care providers are just a few of the variety of reasons that can contribute to these national differences (Andersson et al., 2019).

Obviously, the reasons for the trends shown can be manifold. While some people might argue that CBT is over-investigated, others might favor a position that CBT reveals robust results, and is thus the best anchor for comparisons with other/new interventions. Not surprisingly, CBT has been frequently used as comparison group in non-inferiority trials (Rief & Hofmann, 2018). The decreasing influence of the more traditional approaches, which have also been surpassed by third-wave interventions (e.g. ACT) also poses several questions. Is this just the regular up and down in dynamic research fields that should be accepted and called “progress”? Especially psychotherapy is a vivid field that can reflect the cultural and attitude changes in societies.

Moreover, the success of psychotherapy as a first line treatment for most mental disorders also changed psychotherapy itself. It should no longer be a luxurious and costly treatment option for a few rich people of societies – given the strong evidence base of several psychological treatments, a responsible health care system has the highly important task to develop strategies on how affordable psychological treatment can be made available to all patients who need it (Corscadden, Callander, & Topp, 2018). This need for better availability of evidence-based treatments increases the pressure to develop economic, fast-acting and easily accessible treatments. Accordingly, attempts on how to provide psychological treatments sufficiently on a community and society level are highly laudable, like the IAPT program in England (Clark, 2018).

Is more research needed in psychotherapy? First, there are still clinical fields where too few studies on psychological treatments are available, e.g. anorexia and dissociative disorders (Zhu et al., 2020). Moreover, it is the continuous competition of approaches that helps to better specify and increase the efficacy of interventions. Trends in psychotherapy research cannot only indicate what is more effective, but also what is more suitable for the current needs in society. For example, the rising availability and use of modern technologies (i.e. the internet and smartphones) has laid the groundwork for the development of eHealth interventions. Bringing these treatments to regular health care increases the number of people who can access and benefit from psychological interventions, and enables the treatment for people who would otherwise not have been able to participate in face-to-face treatments (e.g. because of long distances to the closest therapist, Andersson & Titov, 2014).

Continuous psychotherapy research is also the basis for continuing the journey of psychotherapy to become an evidence-based part of most national health care systems. First, there are several clinical conditions for which only very few psychological treatments can be considered as evidence-based (such as in schizophrenia, obsessive-compulsive disorder, insomnia). It was a huge success for the field of psychotherapy to show that specific psychological treatments are effective in psychosis (Lincoln et al., 2012; Lincoln & Pedersen, 2019), even if no concurrent medication is used (Morrison et al., 2018). Others found better effects for depression-specific interventions compared to plausible, but disorder-unspecific treatments (Schramm et al., 2017). These are just a few examples confirming the potential of current psychotherapy research. Further, the more treatment studies we have for one condition, the better we can predict expected treatment outcome. This allows us to compare new study results with these anchors of expected effects. And even if many comparison studies have not revealed significant differences between distinct interventions, some studies did (Poulsen & Lunn, 2014; Schramm et al., 2017; Simon et al., 2021). All these studies on psychological treatments provide important information for scientists, clinicians and stakeholders of health care systems alike.

Some people argue that psychotherapy research is just a reflection of the feasibility of some interventions being used in clinical trials, which does not mirror the necessity of these interventions in clinical practice (Bohart, 2000). This can be considered right and wrong. However, before implementing insufficiently evaluated interventions in a national health care system, studies using controlled designs and valid outcome measures are necessary to prove their efficacy and thus justify their implementation.

Our analysis has some specific limitations, such as the focus on one database (WoS) and on randomized clinical trials. We did not aim for a complete representation of all trials investigating all specific treatments, but rather aimed to find indices of current treatment trends. Whatever approach is selected to find these indices, it always has its specific characteristic and limitations, therefore, we consider our limitations also as a characteristic of this analysis. Others might follow with similar analyses, but using other data sets and other inclusion criteria. For instance, a more hierarchical approach could also be suitable to reveal insights in research developments, starting with a major category (e.g., CBT), and continuing with more detailed analyses (e.g., eHealth interventions using CBT). Of special note is our limitation to RCTs. We are aware that much more clinically relevant studies exist, such as process-oriented trials, qualitative research, effectiveness trials with mere pre-post-comparisons etc. It was our specific aim to focus on RCTs, as this is the study design with the most influence on treatment guidelines (e.g., guidelines of National Institute for Health and Care Excellence NICE; Arbeitsgemeinschaft wissenschaftlich-medizinischer Fachgesellschaften AWMF). However, we agree that the development of psychotherapy research from more traditional approaches investigating one treatment package for one clinical condition, to more process-based treatments and competence-based training of psychotherapists will have consequences for adequate trial designs and thus future trends in psychotherapy research (Hofmann & Hayes, 2019; Rief, 2021).

A further unique part that defines the limitations of our approach is the selection of psychological treatments, and the selection of search terms. We focused on comparing three major treatment approaches with a long history (psychodynamic, systemic and CBT) with more recently developed and outlined approaches, such as ACT, schema therapy, mindfulness, emotion-focused therapy and IPT. Furthermore, we wanted to know what role eHealth developments play in relation to these interventions that are typically provided face-to-face. Of course, this method left many developments unconsidered, such as Unified Protocol approaches, EMDR, or CBASP, to name a few. However, using a comprehensive list of search terms and specific techniques would have been nearly impossible, particularly for CBT techniques. Therefore, we decided to limit this search to major techniques, hereby neglecting further trials that focus on CBT techniques such as stimulus control, habit reversal, or DBT. Further, especially considering this large field of CBT trials, we do not expect substantial differences in percentages if a more inclusive approach would be selected.

Finally, such a database invites to do more detailed analyses on further variables, such as sample sizes, diagnostic unities, study quality, comorbidity, to name just a few. For this first article, this was beyond the scope of the paper.

To conclude, our study confirms the dynamic character of the field of psychotherapy research, with continuously increasing numbers of published trials. It further strengthens the note that the field is not constant, but in continuous change. While new interventions conquer more and more parts of the field, others are losing their representation. Unless we have evidence for negative effects due to these developments, they are primarily to be interpreted as dynamic changes in a developing field. With these changes, challenges for health care systems become evident: How can new developments be considered and eventually included in notoriously conservative health care systems? Our active field of psychotherapy research has shown that it provides specific, evidence-based treatments for most mental disorders, and accordingly, the most powerful and evidence-based treatments should be made available to all patients who need it.

## Supplementary Materials

Further details about the search process and origin of studies are presented in the Supplementary Materials (for access see Index of Supplementary Materials below).

10.23668/psycharchives.6892 Supplement 1 Supplementary materials to "Selected trends in psychotherapy research: An index analysis of RCTs"

RiefW.
KoppM.
AwarzamaniR.
WeiseC.
 (2022). Supplementary materials to "Selected trends in psychotherapy research: An index analysis of RCTs"
[Additional information]. PsychOpen. 10.23668/psycharchives.6892
PMC966742336397942
