# Supplementary material for: Selected Trends in Psychotherapy Research: An Index Analysis of RCTs
Source: Clin Psychol Eur. 2022 Jun 30;4(2):e7921. doi: 10.32872/cpe.7921 (PMC9667423; doi:10.32872/cpe.7921)
Supplement: Supplement 1 [file cpe-04-7921-s01.pdf]

Supplementary Material to the article:

Rief, W., Kopp, M., Awarzamani, R. & Weise, C. (2022). Selected Trends in Psychotherapy Research: An Index Analysis of RCTs. ***Clinical Psychology in Europe.***
https://doi.org/10.32872/cpe.7921

Table 1: Numbers of included studies per treatment and year

Year	Mind-full-ness	ACT	EFT	eHealth	IPT	CBT	Psycho-dynamic	Schema Therapy	Systemic Therapy
2010	1	3	0	12	3	43	3	0	2
2011	0	4	1	21	7	77	2	0	3
2012	0	7	0	20	5	72	8	1	2
2013	1	3	0	29	2	96	8	2	1
2014	0	5	1	31	4	108	7	1	2
2015	1	6	0	28	3	126	5	3	3
2016	3	6	2	33	4	128	4	1	2
2017	3	7	1	42	6	133	9	0	1
2018	5	11	2	35	3	154	4	1	2
2019	7	9	2	43	5	157	3	1	3
Total	21	61	9	294	42	1094	53	10	21

Note. **Database**: Web of Science (WoS)

Selected Web of Science categories:

- psychiatry
- health care sciences and services
- psychology, clinical
- medical informatics
- medicine general and internal
- medicine research and experimental
- public, environmental and occupational health
- clinical neurology
- psychology
- neurosciences
- multidisciplinary sciences
- rehabilitation
- psychology, multidisciplinary
- substance abuse
- pediatrics
- behavioral sciences
- psychology, developmental
- social sciences, biomedical
- critical care medicine
- education and educational research
- psychology, experimental
- family studies
- psychology, applied

Search Terms in the **Title** of the article:

All searches:

- Selection to specific clinically-relevant categories, see main text
- “RCT” OR “randomi*” (according to the CONSORT criteria, all randomized clinical trials should include the terms “RCT” or “randomi*” in the title (Schulz et al., 2010))

Specific searches:

- cognitive behavior therapy: “CBT” OR “cognitive” OR “cognitive behavio*” OR ”exposure therapy” OR “behavioral activation”
- internet-based psychological treatments: “eHealth” OR “mHealth” OR “internet”
- psychodynamic treatments: “psychodynami*” OR “psychoanaly*” OR transference” OR “mentali*”
- Mindfulness-based Interventions: “mindful” OR “MBCT” OR “MBSR”
- Acceptance and commitment therapy: “ACT” OR “commitment” OR “commit*”
- Interpersonal therapy: “IPT” or “interpersonal”
- systemic therapy: “systemic* therapy” OR “family therapy”
- schema therapy: “schema*”
- emotion focused therapy (EFT): “EFT” OR “emotion-focus*” OR “ef therapy” (Title)
